# Paradoxical Interplay of Viral and Cellular Functions

**DOI:** 10.3390/v3030272

**Published:** 2011-03-15

**Authors:** Esteban Domingo

**Affiliations:** 1 Centro de Biología Molecular Severo Ochoa (CSIC-UAM), C/ Nicolás Cabrera, 1, Cantoblanco, Madrid 28049, Spain; 2 Centro de Investigación Biomédica en Red de Enfermedades Hepáticas y Digestivas (CIBERehd), Barcelona 08036, Spain; E-Mail: edomingo@cbm.uam.es; Tel.: +34-91-1964540; Fax: +34-91-1964420

**Keywords:** RNA virus, host-virus interaction, coevolution

## Abstract

Some cellular editing functions can restrict the replication of some viruses but contribute to completion of the life cycle of others. A recent study has identified an isoform of the adenosine deaminase acting on RNA type 1 (ADAR 1) as required for embryogenesis, and as a restriction factor for a number of important RNA virus pathogens [1]. The dual implication of key cellular functions in the innate immunity against viruses, or, paradoxically, as mediators of virus replication is interpreted in the light of the concept of virus-host coevolution and tinkering proposed for general evolution by François Jacob decades ago.

## 1. Introduction

A recent publication by M.B. Oldstone and colleagues at the Scripps Research Institute of La Jolla has documented that one of the isoforms of the adenosine deaminase acting on RNA type 1 (ADAR 1) is a restriction factor for replication of measles virus (MV), other members of the Paramyxoviridae family, and influenza virus, but not for other RNA viruses such as lymphocytic choriomeningitis virus (LCMV), vesicular stomatitis virus or reovirus [1]. The *ADAR 1* gene encodes two proteins spanning multiple exons, with a critical difference being in the presence of either exon 1A or 1B, expressed from two different promoters. Exon 1A is under the control of IFN-α and generates the p150 isoform while expression from 1B is constitutive and gives rise to the p110 isoform. In an elegant approach, a targetting vector was designed for the specific elimination of the IFN-inducible p150 isoform of ADAR 1, leaving intact the constitutive expression of the p110 isoform. It was known that inactivation of ADAR 1 leads to embryonic lethality through uncontrolled apoptosis and liver disintegration [2,3]. Mice heterozygous for the targeted *ADAR 1* were generated and interbred to obtain embryos in which the two copies of the genomic region encoding p150 were disrupted. Embryos which were negative for p150 could not complete development but they were the source for the mouse embryo fibroblast (MEF) cells for further study. Wild type and p150-deficient MEF cells were engineered to express the MV receptor signaling lymphocyte activation molecule (SLAM or CD150). Comparison of infectious progeny production and cytopathology indicated an anti-MV activity of the p150 isoform of ADAR 1. Thus, the main methodological novelty of the study, the selective elimination of p150, demonstrated that the absence of this isoform was associated with embryonic lethality and susceptibility to infection by MV and other paramyxoviruses [1]. The results open the way to studies on the function of ADAR 1 *in vivo*, as well as to investigations on viral proteins that may antagonize the ADAR 1 antiviral activity.

## 2. Increasing Functional Scope of RNA Editing

The ADARs are a family of adenosine (A) to inosine (I) editing enzymes that act on double-stranded RNA, encoded by three genes (*ADAR* 1–3) identified in mammals (reviewed in [4]). RNA editing was discovered in trypanosomes and involved insertion or deletion of uridine residues in their mitochondrial mRNA, modifications that gave rise to the synthesis of several proteins [5,6]. One of the biological consequences of editing acting on protein-coding RNA is that multiple proteins can be expressed from the same coding region, with minimal chemical alteration of the RNA. Very often the proteins expressed from edited and non-edited cognate RNAs perform different biological roles, thus expanding the functional capabilities of a given coding region. Mechanistically, at least two types of editing reactions have been distinguished: those that introduce insertions or deletions (for example by polymerase slippage or stuttering), and those that convert one nucleotide type into another through hydrolytic deamination of nucleotides. Editing reactions can be highly selective for one or a few residues or nearly random in that they may target multiple residues in coding or non-coding RNAs. Additional editing proteins are those in the APOBEC family, a term that refers to the apolipoprotein B mRNA editing complex. A specific cytidine (C) to uridine (U) deamination of the apolipoprotein B mRNA gives rise to a truncated form of the protein, and the two proteins that are generated as a result of this editing event display different roles in lipid metabolism [7,8]. The APOBEC3G can act as a restriction factor for retroviruses through hypermutagenesis of the viral genome, a reaction which in the case of HIV-1 is counteracted by the virus-coded protein Vif.

The ADARs were discovered in *Xenopus laevis* embryos as a double-stranded RNA-unwinding activity, later identified as an A→I deaminase [9,10]. The three forms of ADAR share deaminase and double-stranded RNA-binding domains, and are highly conserved in vertebrates. A related family of proteins are the ADATs, or adenosine deaminases acting on tRNA, that catalyze A→I editing in tRNAs. The enzymes cytidine deaminases acting on mononucleotides may have been the evolutionary ancestors of ADATs, and the latter, in turn, the precursors of ADARs [11]. An interesting possibility is that the *ADAR* genes evolved from the *ADAT* upon acquisition of a region that encodes a double-stranded RNA-binding domain. This would be yet another illustration of modular evolution of cellular proteins, a form of functional expansion and diversification prominent also in virus evolution.

The A→I RNA editing mediated by ADAR proteins affects mainly non-coding RNA sequences: untranslated regions, introns, repetitive RNA elements, and precursors of some microRNAs (miRNAs), among other targets [4]. Double-stranded RNA regions of more than 20 base pairs (bp) folded within the same RNA molecule or involving two different molecules can be substrates for the ADAR enzymes. While short RNAs of around 20 bp tend to be edited selectively, double-stranded RNAs of more than 100 bp tend to be abundantly deaminated, leading to the hypermutated viral RNAs such as in the variant forms of MV found in the brain of patients afflicted with subacute sclerosing panencephalitis (SSPE) and other MV-related neurological diseases. The number of cellular targets of ADAR reflects an exuberant multifunctionality of this protein family, and suggests that this editing mechanism may boost functional capacities of non-coding RNAs and short regulatory RNAs whose roles in the biology of the cell remain largely unsolved. In addition, the ADAR proteins themselves can have direct functional roles such as in nuclear export complexes of some double stranded RNAs [12]. ADAR-editing reactions have been associated with the generation or abolition of splice donor and acceptor sites, with regulation of endogenous short interfering RNAs (esiRNAs), and with siRNA-and miRNA-mediated gene silencing, among other activities. Gene silencing which is dependent on miRNAs plays an important role in apoptosis, differentiation and development [13]. It is tempting to speculate that through perturbations of miRNA-dependent functions, the absence of the p150 isoform of ADAR 1 can lead to embryonic lethality [1].

Editing activities can promote viral replication, and not only in the form of hypermutated MV genomes found in SSPE brains. Hepatitis delta virus (HDV) has a negative sense RNA genome (of opposite polarity to that needed for expression of the encoded proteins) and replicates via a positive sense RNA termed anti-genome. HDV requires hepatitis B virus (HBV) as helper virus that supplies the surface protein. Editing by ADAR occurs in the nucleus and allows synthesis of two forms of the protein encoded by HDV (long, L and short, S) from the same m-RNA. The S version is necessary for genome replication and L for virion assembly. Their expression is temporally regulated since L may inhibit S-mediated viral replication. Part of the benefits of IFN-α against HDV infection may be due to an increase of L HDV protein expression, resulting in a decrease of viral replication. Thus, cellular editing functions that play key functional roles in the cell can restrict the replication of some viruses, but can also contribute to the completion of the life cycle of other viruses.

## 3. The Paradoxical Exploitation of Cellular Functions for Virus Restriction or Virus Multiplication: Cell-Virus “Co-Evolution and Tinkering”

Several cellular functions may modulate the susceptibility of cells to viral infection, through a variety of mechanisms referred to as innate immunity. Among classical examples are the effects of murine proteins Fv1 and Fv4, which are the products of expression of endogenous retroviruses, on the retroviral life cycle. Fv1 blocks the entry into the nucleus of retroviral preintegration complexes, and Fv4 acts as a non-functional env protein that competes with the authentic viral env protein for receptor binding [14]. Other examples are the tripartite motif protein 5α (TRIM5α)- and zinc-finger antiviral protein (ZAP)-mediated restriction of retroviral functions, or the APOBEC family of proteins that may limit viral infection through hypermutagenesis of viral genomes.

Editing may have promoted evolutionary adaptations of viral genomes. The polymerase of the Paramyxovirinae (the subfamily of Paramyxoviridae that includes MV) stutters in a controlled manner to edit the viral phosphoprotein gene mRNA. It has been suggested that these viruses may have evolved a genome of polyhexameric length (the so-called “rule of six”) to prevent the deleterious effects of illegitimate or uncontrolled editing that may result in hypermutagenesis [15]. Enhanced mutagenesis is exploited by cellular systems as a defence against invading molecular parasites. The “repeat induced point mutations (RIP)” system operates in some filamentous fungi to produce mutations in genetic elements with repeated DNA copies that penetrate into the cells [16]. The mechanisms of innate immunity based on the introduction of mutations with high frequency, leading to inactivation of viruses or other invading parasitic genomes, lend support to the concept of lethal mutagenesis or virus entry into error catastrophe (extinction of viruses through an excess of mutations), currently under investigation as an antiviral strategy [17,18].

Yet, essential cellular proteins are, paradoxically, also key players in the life cycle of viruses. A classical example is the participation of host proteins in viral polymerases. The first documented case was the RNA-dependent-RNA polymerase (RdRp) of *E.coli* bacteriophage Qβ. This is a multi-subunit enzyme with one phage-coded protein and three bacterial proteins: the ribosomal protein S1, and translation elongation factors EF-Tu and EF-Ts [19]. Other viral RdRps also require host proteins for activity (actin or tubulin for some paramyxoviruses or poly (rC)- and poly r(A)-binding proteins for picornaviruses [20]). Virologists are well aware of many additional cellular proteins, intracellular membranes and other cell components and compartments that upon infection are efficiently diverted from their standard role in the cell to the benefit of a virus.

How can this paradoxical interplay of cellular functions participating either in innate cellular immunity or in virus replication be interpreted from an evolutionary point of view? In terms of ADAR, why the same editing functions can be a restriction factor for MV and at the same time promote genetic changes that result in virus persistence in the brain? Hypermutation enhanced MV cell-to-cell spread within the infected host eventually allowing the virus to invade the CNS, as opposed to normal MV which spreads by budding particles. Almost one quarter of a century ago, François Jacob wrote an influential article entitled “Evolution and tinkering” [21], based on a lecture that he delivered at UC Berkeley in 1977. The article was a statement of the problems of complexity as they apply to biological systems, a concept that finds an increasing impact in current biology. Jacob compared the action of natural selection to the activity of a tinkerer rather than to that of an engineer, and reviewed multiple lines of evidence that evolution is far from perfect, and that there is no engineer to guide it. To introduce this point, he referred to 16th century illustrations displaying odd monsters consisting of chimeric creatures possessing anatomical parts that belonged to different biological species. Examples of functions that result from evolutionary tinkering are found in all domains of biology, from metabolism to brain function. In the long history of coevolution that viruses and cells have probably experienced in our biosphere, the astonishing complexity of virus host-interactions must have been also the result of tinkering. All the evidence points towards cells and viruses having taken advantage, as needed, of what existed at the corresponding stage of evolution. Using what was available to construct what was possible must have led to monstrous protein complexes with cellular proteins participating in the killing of the cells that originated them, or the same cellular proteins used either to combat or help viruses. In the case of SSPE, ADAR-1 editing provided a short-term, accidental selective advantage for the virus to persist transiently in the human brain. Tinkering had to respond to the immediate needs of the biological systems at play, with the only filter of trial and error. In this view, cellular editing enzymes may have served either to restrict the replication of a virus or to create a form that survives in the human brain. In the race for virus and cell survival, tinkering mediated the survival of cells or of their virus parasites, as blindly dictated by natural selection at any given time. However, a detailed understanding of the mechanisms that evolution invented in an improvised fashion to limit the replication of viruses may inspire means of human intervention to control viral infections.
